# Novel Therapeutic Approach for Inhibition of HIV-1 Using Cell-Penetrating Peptide and Bacterial Toxins

**DOI:** 10.4172/2155-6113.1000737

**Published:** 2017-10-23

**Authors:** Steven Samuels, Zainab Alwan, Marceline Egnin, Jessie Jaynes, Terry D Connell, Gregory C. Bernard, Toufic Nashar

**Affiliations:** 1 Environment and Nutrition Sciences, Faculty of Agriculture, Tuskegee University, Tuskegee, Alabama, USA; 2 Department of Life and Earth Sciences, Perimeter College at Georgia State University, 555 North Indian Creek Drive, Clarkston, USA; 3 Department of Pathobiology, College of Veterinary Medicine, Tuskegee University, Tuskegee, Alabama, USA; 4 Department of Community Health, Institute of Medical Technology, Middle Technical University Baghdad, Iraq; 5 Department of Microbiology and Immunology, The Witebsky Center for Microbial Pathogenesis and Immunology, Buffalo, New York, USA

**Keywords:** HIV-1, Gag p24, Peptides, Bacterial toxins, Pseudovirus, Drugs, Enterotoxins

## Abstract

Despite advancements in our understanding of HIV-1 pathogenesis, critical virus components for immunity, vaccines trials, and drugs development, challenges remain in the fight against HIV-1. Of great importance is the inhibitory function of microbicidal cell penetrating peptides and bacterial toxins that interfere with production and neutralize infection of HIV-1 particles. We demonstrate that the neutralizing activity of a cationic 18 amino acids peptide, is similar to a broadly neutralizing human antibody, and inhibits production of two HIV-1 strains in human cell lines. Pretreatment of cells with bacterial toxins or toxoids derived from enterotoxigenic *E. coli*, boost subsequent activity of the peptide against HIV-1, to inhibit simultaneously production and infection. The synthetic peptide crosses the cell membrane into the cytoplasm and nucleus. *In vitro* analysis of a possible target for this peptide revealed specific binding to recombinant HIV-1 gag p24. This is the first demonstration of a synergy between bacterial toxins and a cell-penetrating peptide against HIV-1.

## Introduction

A number of strategies have been designed to combat the development of AIDS in persons infected with the human immunodeficiency virus (HIV-1), including testing prototype vaccines and treatment with antiviral drugs. However, several hurdles are to overcome in HIV-1 vaccine development [1–5]. Additionally, antiretroviral drug therapy has serious clinical consequences due to development of resistance by the virus [6]. Moreover, the constant mutational property of the HIV-1 RNA and shielding of proteins on the surface of the virus by glycans poses a challenge for development of antibody therapy, although more recent antibody-like inhibitors may look promising [7]. Hence, development of preventative agents against HIV-1, among others, is a high priority. Of great importance is the inhibitory function of microbicidal cell-penetrating peptides and bacterial toxins that interfere with production and neutralize infection of HIV-1 particles. The advantages gained from this approach would be the reduced ability of the HIV-1 virus to develop resistance due to the small size of the peptides, and interference with HIV-1 infection and replication by the peptide and toxins. Many peptides such as defensins, indolicidin, polyphemusin, and melittin, have activity against viruses including HIV-1, herpes simplex virus, influenza A virus, and vesicular stomatitis virus [8–10]. They also have the ability to enter cell membranes without aide [9,11]. Most of these are positively charged, self-internalize, and have the ability to reach the cytoplasmic and/or nuclear compartments [11]. Moreover, peptides are natural products, have low toxicity and immunogenicity. They also inhibit protein subunits or protein-protein interactions due to their small size.

To infect cells, HIV-1 uses a combination of receptors that undergo conformation changes allowing the virus to enter the cell. Among these receptors is a primary CD4 and chemokine co-receptors CXCR4 and CCR5 [12]. The *gag* gene, one of three genes involved in HIV-1 particle assembly encodes HIV-1 gag proteins. Gag protein is formed initially as a polyprotein precursor, Pr55gag, which is cleaved off during or after virus budding, into mature proteins p17 (matrix), p24 (capsid, CA), p7 (nucleocapsid), and p6 [13]. In cells, the capsid protein p24 is involved in virus assembly, maturation, and virus post-entry events [14]. Hence, gag p24 mutations impose a fitness cost on viral replication making it a good target against HIV-1 [13]. The *Env* gene encodes the viral envelope (Env) glycoproteins, which play a critical role in virus entry. The *pol* gene encodes enzymes essential for virus replication in the infected cell.

Besides cationic peptides, bacterial toxins appear to interfere with HIV-1 infection. Pertussis toxin (PTx) is an oligomeric protein that affects HIV-1 replication in peripheral blood mononuclear cells at both entry and post-entry levels [15]. Additionally, cholera toxin (CT) from Vibrio cholera reduces HIV-1 infection in the human colorectal cell line HT-29 [16]. Our previous work characterized the function and biological properties of *E. coli* heat-labile enterotoxins. Type 1 (LT-I) and type 2 (LT-II) human *E. coli* enterotoxins are composed of an A and a pentameric B-subunit. LTs bind, by their non-toxic B subunits (LT-IB and LT-IIB), to cell-surface ganglioside receptors, which are ubiquitously found on the surfaces of mammalian cells. LTs modulate cellular responses by capping of the membrane receptor generating signal transduction that alters cytokine production and level of expression of surface markers on antigen presenting cells, and T cells. The toxicity of LTs arises from their stimulation of adenylate cyclase, resulting in elevation of cAMP, and subsequent imbalance of electrolytes. As such, the toxins and their non-toxic B-subunits exert profound effect on cell biological processes following binding to their respective receptors (ganglioside GM1 for LT-I, LT-IB; ganglioside GD1-b for LT-IIa, LT-IIaB) [17–20].

Nevertheless, no reports investigated the synergistic effects of toxins and therapeutic peptides Here, we report the activity of an 18 amino acid cationic synthetic peptide on production and infection of two strains of HIV-1 pseudoviruses (DH12 and SF162), in human cell lines. This peptide has an identical sequence to a peptide previously shown to neutralize Feline Immunodeficiency Virus (FIV) [21], except for an additional Methionine at its N-terminus, and is currently investigated in development of plant-based therapeutic approach against HIV-1 [22]. Additionally, we examined possible synergistic activity of the oligomeric toxins and toxoids from enterotoxigenic *E. coli*, with the peptide, in blocking HIV-1 production and infection.

## Materials and Methods

### Cell lines

The human TZM-bl indicator cell line that expresses CD4, CCR5 and CXCR4 was obtained through the NIH AIDS Reagent Program, Division of AIDS, NIAID, NIH: TZM-bl from Dr. John C. Kappes, Dr. Xiaoyun Wu and Tranzyme Inc. [23–27]. The human HK293T cell line (ATCC) was used for virus production. Human mammary epithelial cell line (HMEC, ATCC) was used for peptide uptake and localization. All cell lines were cultured in high glucose Dulbecco’s Modified Eagle’s Medium (DMEM) containing L-glutamine, sodium pyruvate, penicillin/streptomycin, and 10% Fetal Bovine Serum (FBS, Sigma, U.S.A).

### Peptide cell uptake and localization

Human mammary epithelial cells (HMEC. ATCC) were seeded in 60-well plates for 24 h. Ten microliters of biotinylated peptide (Polypeptide Laboratories, U.S.A) dissolved in Hanks Balanced Salt Solution (HBSS) was added at final concentration of 0, 1, 5 and 10 μM, for 2 h, at 37°C. Wells were rinsed with HBSS. After rinsing with HBSS, cells were fixed with cold methanol for 10 min., and rinsed with 20 mM Tris, pH approx. 7.4, and 0.9% NaCl (TBS). Plates were incubated for 60 min. at room temperature (R.T.) with a 1/500 dilution of streptavidin-Alexa 488 (Sigma, U.S.A), rinsed with TBS 3 × 5 min. and then stained 5 min. with DAPI solution, and finally rinsed with TBS. Cells were mounted on slides and examined by fluorescence microscope. Depending on peptide concentration, scoring included weak, intermediate, or strong signal.

### HIV-1 pseudovirus production

The protocol for virus production and neutralization was followed essentially as reported previously [28], with some modifications for the peptide experiments. The 293T cells were seeded at 10^4^ cells/ml, in 12-well plates, and incubated overnight, at 37°C. Cells were transfected with SF162 gp160 (NIH AIDS Reagent Program, Division of AIDS, NIAID, NIH: pCAGGS SF162 gp160 from Drs. L. Stamatatos and C. Cheng-Mayerand) [29–31]. The cells were also co-transfected with a backbone pSG3 env plasmid (NIH AIDS Reagent Program, Division of AIDS, NIAID, NIH from Drs. John C. Kappes and Xiaoyun Wu: pSG3 env) [32,33]. The DH12 virus was produced from a backbone plasmid [34]. The 293T cells were transfected with DH12 and pSG3 env plasmids. To generate the “virus complex”, 0.5 μg of each backbone plasmid was mixed with an equal amount of pSG3Δenv, 3 μl transit 20/20 (Mirus Bio, U.S.A), and Optimum medium (ThermoFisher, U.S.A) to 100 μl total volume. The complex was incubated at R.T., for 30 min. The virus complex was then added dropwise to the cells. Control wells included cells without the virus complex. Plates were incubated for an average of 5 h, at 37°C. The medium was removed, and replaced, after which cells were incubated at 37°C for additional 48 h. Viruses were then harvested in the supernatants, and stored at −80°C. The amount of virus produced was quantitated in luminance units after infection of the indicator TZM-bl cell line, as described below.

### Neutralization of HIV-1 pseudoviruses by 10E8 mAb

A broadly neutralizing HIV-1 antibody 10E8 against all major subtypes (NIH AIDS Reagent Program, Division of AIDS, NIAID, NIH: Anti-HIV-1 gp41 Monoclonal (10E8), from Dr. Mark Connors) [35]. 10^4^ TZM-bl cells/well were incubated in 96-well plates for 1–2 h. To enhance virus entry, the cells were pretreated with 25 μg/ml DEAE Dextran, as previously described [34]. Viruses produced in 293T cells (as above) were serially diluted (5x) with and without a decreasing concentration of 10E8 antibody. Control wells included cells incubated with virus without the antibody (virus control, VC), and cells without treatment (cell control, CC). Plates were incubated for 48 h, after which supernatant was removed. 60 μl of sterile diH2O and an equal volume of Bright-Glo (TM) Luciferase substrate (Promega, U.S.A) was added to the cells. Aliquotes of 100 μl cell lysate was then transferred to black solid microplates (Sigma, U.S.A). Plates were read (Synergy 2 Multi-mode microplate reader) to determine the luciferase activity which is directly proportional to the number of infectious virus particles present in the initial inoculum, over a wide range of values. Percent neutralization was calculated based on the following formula: 1−[(treatment samples average)/(VC−CC)] × 100. The samples average represents average of readings from each virus dilution corresponding to each antibody concentration.

### Neutralization of HIV-1 pseudoviruses by the peptide

An 18 amino acids peptide (MFKLRAKIKVRLRAKIKL) was synthesized at >80% purity (Life Technologies, U.S.A). To determine the inhibitory dose of the peptide, viruses were produced in 293T cell supernatants, TZM-bl cells were seeded, as described above. 25 μl of supernatant containing virus was serially diluted in 100 μl cell culture medium (5x) and added to the cells. At the same time, the peptide was serially diluted (10x) from a stock at the indicated concentrations in a final volume of 100 μl, and added to the cells. Plates were incubated for 48 h at 37°C.

### Effects of the peptide on HIV-1 production, neutralization, and both production and neutralization

For peptide effects on virus production, 293T cells were seeded in plates for 24 h, transfected with plasmids encoding the virus envelope and the backbone for each virus. Cells and plasmids were incubated for 3 h, at 37°C (time required for the plasmids to enter cells). The peptide was serially diluted (3x) and added to cells. Viruses were then harvested in the culture supernatants, after 48 h. For peptide effects on neutralization of HIV-1 strains, viruses produced in 293T cells supernatants without the peptide were mixed with serial dilution of the peptide, and the mixture was added to TZM-bl cells, and incubated for 48 h. For the concurrent effects of the peptide on virus production and neutralization, 293 T cells were initially transfected with the virus envelope and the backbone plasmids, and incubated for 3 h, at 37°C. Nine microM of the peptide was then added to the cells. Plates were incubated for 48 h, and supernatants containing virus were harvested. Aliquotes of 25 μl of each supernatant from 293T cells were mixed with a serially diluted peptide (3x), and added to the indicator TZM-bl cells. Plates were incubated for 48 h, at 37°C.

In all of the stated protocols above, plates were read to measure the reflective light units in TZM-bl cells, which were converted to “%virus inhibition”. These were calculated relative to a positive control, which consisted of cells (293 T or TZM-bl) incubated without the peptide.

### Effects of a mixture of peptide and bacterial toxins on virus production and infection

For the peptide and toxins or toxoids effects on virus production and infection, 293T cells were seeded in plates for 24 h, and transfected with the plasmids encoding the virus envelope and the backbone plasmid for each virus, as described above. After 3 h incubation at 37°C, a mixture consisting of 9 μM peptide and each of the bacterial toxin or toxoid (LT-I, 1 μg/ml; LT-IIa, 1 μg/ml; LT-IB, 2 μg/ml; LT-IIaB, 2 μg/ml) was added to the cells. Plates were incubated for 48 h, at 37°C. Supernatants containing each virus were harvested. TZM-bl cells were pretreated with the bacterial toxins or toxoids, at the indicated doses above, and incubated for 2 h. Virus-containing supernatants were then serially diluted (5x) in a mixture with the peptide at the indicated concentrations above. The mixture was then added to the cells. Plates were incubated for 48 h, at 37°C. “Percent virus inhibition” was calculated relative to a positive control consisting of cells incubated with the virus without treatments.

### Evaluation of bacterial toxins and toxoids effects on viability of TZM-bl cells

TZM-bl cells were seeded at 10^4^ cells/well for 48 h. Two microgram/ml LT-IB or LT-IIaB, or 1 μg/ml LT-I and LT-IIa were added to the cells, and plates were incubated for 24 h. Following incubation, cells were harvested, washed, and mixed with 40 μg/ml propidium iodide. Acquisition was performed by flow cytometry (Becton Dickenson), and analysis by FlowJo software (Tree Star, OR).

### ELISA for interaction of peptide with HIV-1 gag p24

A 96-well ELISA plate was coated with 10 μg/ml of the peptide diluted in carbonate/bicarbonate buffer, PH 9.6. Wells were blocked with 1% BSA for 1 h, at 37°C. Recombinant HIV-1 gag p24 was expressed and purified from *E. coli*, as described before [36,37]. Serial 2x dilution of gag p24 was added to the wells. Wells coated directly with 10 μg/ml gag p24 without the peptide acted as a positive control. Wells coated with JC41N peptide in the absence of gag p24 acted as a negative control. Plate was incubated for 2 h at 37°C. Following incubation and washings, mouse monoclonal anti-gag p24 antibody (ProSpec, U.S.A) was added, at 1/10,000 in TBS/Tween-20/0.2% BSA, and incubated for 2 h at RT. Following the washings, alkaline phosphatase conjugated goat anti-mouse IgG (Fab) (Sigma, U.S.A) diluted in TBS/Tween 20/BSA was added at 1/25000, and incubated overnight at 4°C. The reaction was developed by addition of alkaline phosphatase substrate (Sigma, U.S.A), and read at 405 nm.

## Results

### Peptide uptake and localization in the cytoplasm and nucleus

**Figure 1:**
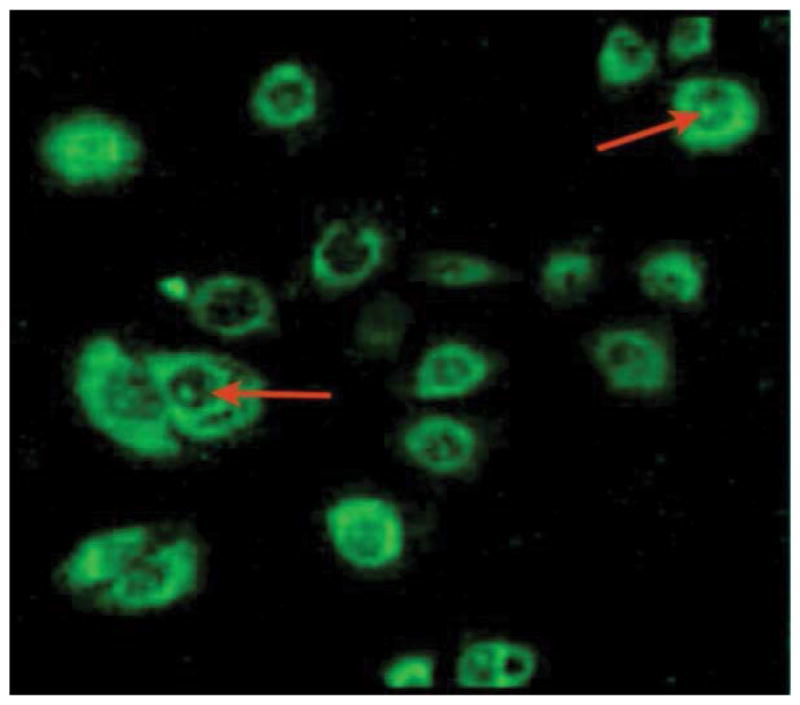
The peptide localizes in the cytoplasm and nucleus after cellular uptake. Human mammary epithelial cells (HMEC) were seeded in plates for 24 h. Biotinylated form of the peptide was added at final concentrations of 0, 1, 5, and 10 μM. Cell were incubated for 2 h, at 37°C. Cells were then fixed with cold methanol and stained with Streptavidin-Alexa 488, followed by DAPI solution for 5 min. Shown is a representation of cells that exhibited strong localization staining of the peptide (5 or 10 μg/ml peptide) in the cytoplasm and nucleus. No staining was present in cells without the peptide.

Cationic peptides enter cells by various mechanisms. To examine whether the 18 amino acid peptide enters cells, human mammary epithelial cell line (HMEC) was incubated with the peptide, which was modified by biotinylation, and probed with streptavidin-Alexa. In the absence of the peptide, there was no fluorescence signal detected (not shown). However, fluorescence signals were present in cells incubated with the peptide at 1, 5, and 10 μM, with stronger localization of the signals in the cytoplasm and nucleus, detected at 5 and 10 μM (Figure 1, representative of 10 μM of the peptide). We conclude that incubation with the 18 amino acid peptide results in its intracellular localization.

### Comparison between Antibody and peptide neutralization of HIV-1 infection

**Figure 2:**
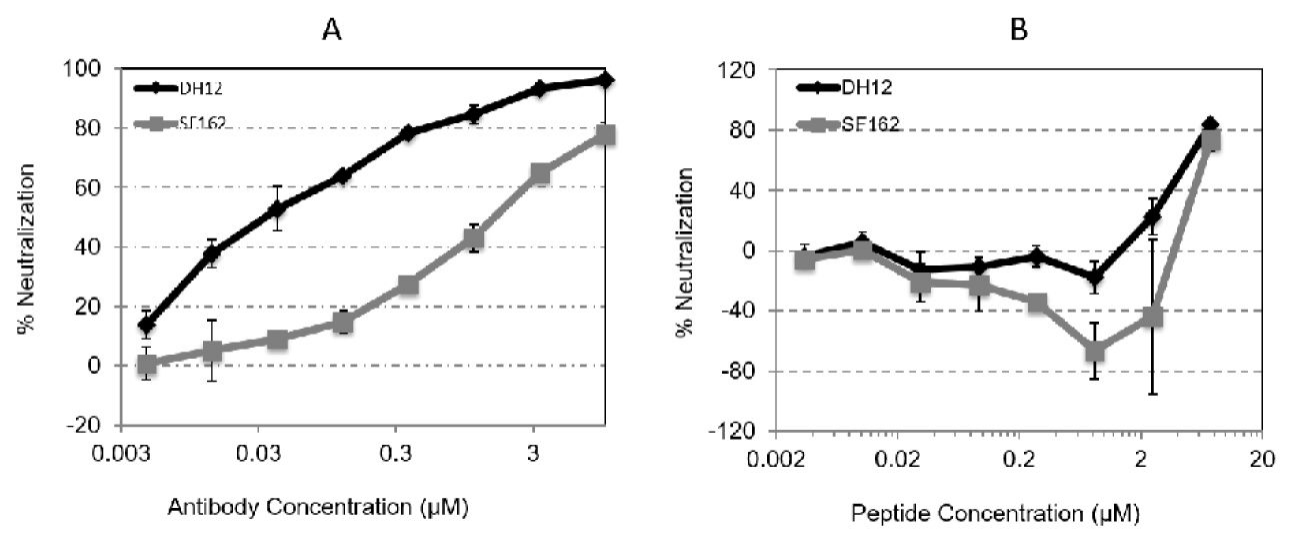
10E8 antibody and the peptide are equally effective at neutralization of HIV-1 strains at the higher doses. DH12 and SF162 HIV-1 strains were produced in 293T cells. Viruses in supernatant from 293T cells were added to the indicator cell line TZM-bl cells with and without increasing concentrations of 10E8 mAb (A), or peptide (B). Cells were then incubated for 48 h, at 37°C. Plates were read to measure the reflective light units, which were converted to percent neutralization (see M&M). The data points represent mean of five replicate wells +/− SD.

We compared neutralization of HIV-1 infection by the peptide to the broadly HIV-1 neutralizing antibody, 10 E8. Neutralization is measured in the reporter cell line TZM-bl as a function of reductions in HIV-1 Tat-regulated luciferase reporter gene expression following a single round of infection with Envelope (ENV)-pseudotyped HIV-1 viruses. To generate HIV-1 strains, namely DH12 and SF162, 293 T cells were transfected with virus backbone plasmid and virus envelope-encoding plasmid. Viruses were harvested after 48 h. To examine neutralization activity, the indicator TZM-bl cells were infected with each virus concomitantly with increasing concentrations of the antibody or the peptide. After 48 h incubation, the amount of luminance in these cells was measured, and correlated to positive and negative controls. Figure 2 shows that there was a gradual increase in the neutralizing capacity of the 10E8 antibody with the increase in its concentration. The antibody was more effective in neutralizing HIV-1 DH-12 compared to SF162, at all doses. It took 10 μM of the antibody to attain maximum neutralizing activities against DH12 and SF162, reaching 78% and 96%, respectively. In comparison, the lower concentration of the peptide up to 2 μM showed no neutralizing activity against SF162, and was minimal against DH12 virus. At concentration of the peptide >2 μM, the neutralizing activity against both viruses was 80% at 7.5 μM. Hence, the peptide and the antibody had similar efficacy at the high dose. Like the 10E8 antibody, the peptide was less effective against SF162 compared to DH12 virus, particularly at the lower concentrations. We conclude that the peptide neutralizing activity against the HIV-1 strains is similar to that of the broadly neutralizing 10E8 antibody.

### The peptide simultaneously inhibits HIV-1 production and infection

**Figure 3:**
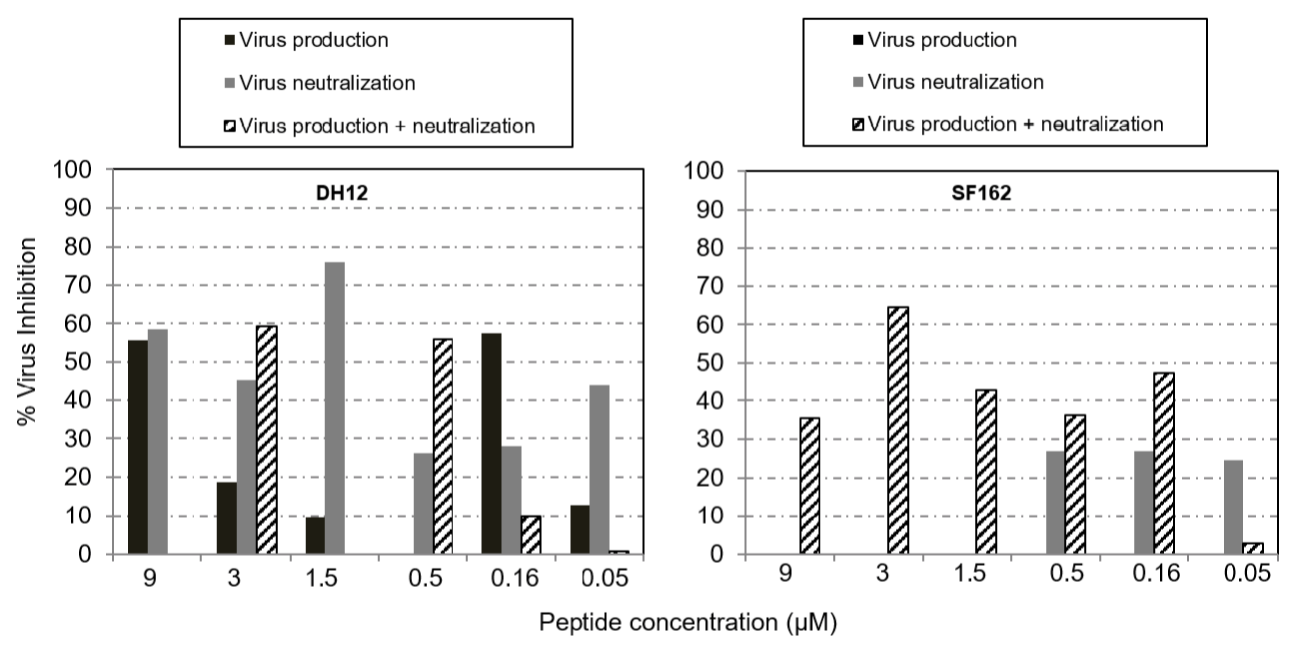
The peptide inhibits HIV-1 production and infection. For peptide effects on virus production, 293T cells were seeded in plates for 24 h, transfected with plasmids encoding the virus envelope and the backbone plasmid for each virus, with and without increasing concentrations of the peptide. The viruses were then harvested after 48 h. For peptide effects on infection, viruses produced in 293T cells without the peptide were added to seed TZM-bl cells with increasing concentrations of the peptide. Cells and peptide were incubated for 48 h. For the simultaneous effects of the peptide on virus production and infection, viruses produced in 293T cells in the presence of 9 μM of the peptide were subsequently treated with increasing concentrations of the peptide before adding the mixture to TZM-bl cells. Plates were incubated for 48 h. The reflective light units were then measured for each virus, which were converted to “percent virus inhibition” relative to a positive control consisted of cells incubated without the peptide. The peptide inhibitory effects on virus production and infection were observed in at least similar experiments.

The previous experiment demonstrated that the peptide or antibody neutralized HIV-1 infection when added to the indicator TZM-bl cells with the virus, which is a later step after production of virus progeny. Activity of the peptide against DH12 and SF162 viruses was examined during and after formation of virus progeny (Figure 3). For effects on production of virus progeny, 293T cells were transfected with each of the viruses’ backbone and ENV-encoding plasmids followed by the peptide. Virus production was verified in the indicator TZM-bl cells. As shown in Figure 3, high activity of the peptide against DH12 virus was evident at 9 μM (55%) and 0.16 μM (57%). The activity against the virus correlated well with the dose of the peptide except 0.16 μM, which was equal to 9 μM. A number of factors could influence the variation in the effective peptide concentration, including peptide cell entry mechanisms influenced by the amount of added peptide, peptide’s entrance rate, and peptide chemical property such as its solubility. In this regard, the 18 amino acid peptide is insoluble in PBS, and had to be diluted in Tris buffer to maximally increase its solubility. While the peptide inhibited HIV-1 DH12 production, it had no anti-viral activity against SF162 virus. This again points out, like with the 10E8 antibody, to the difficulty in inhibiting this HIV-1 strain. These results indicate that the peptide interferes with production of HIV-1 DH12.

To examine the neutralizing activity of the peptide against HIV-1 infection (Figure 3), viruses produced without the peptide in 293T cells supernatants, were mixed with an increasing amount of the peptide before infection of TZM-bl cells. Highest neutralizing activity against DH12 virus was present at 9 μM and 1.5 μM (58% and 76%, respectively). In comparison, the peptide activity against SF162 virus was only evident at the lower doses of 0.5, 0.16, and 0.05 μM, and maximal inhibition was 26% only.

We also examined the simultaneous peptide activity against HIV-1 production and infection (Figure 3). Due to the peptide high activity against HIV-1 DH12 production at 9 μM, this dose was chosen to treat 293T cells. Following production of the viruses in presence of the peptide, the activity of a mixture of the supernatants and a serially diluted peptide was evaluated in TZM-bl cells. Inhibition of production and infection of DH12 virus was evident at 0.5 μM and 3μM and (56 and 59%, respectively). Importantly, all doses of the peptide inhibited the HIV-1 SF162 strain (maximal 76% at 3 μM). We conclude that the 18 amino acid peptide has a broad activity against production and infection of the HIV-1 DH12 and SF162.

**Figure 4:**
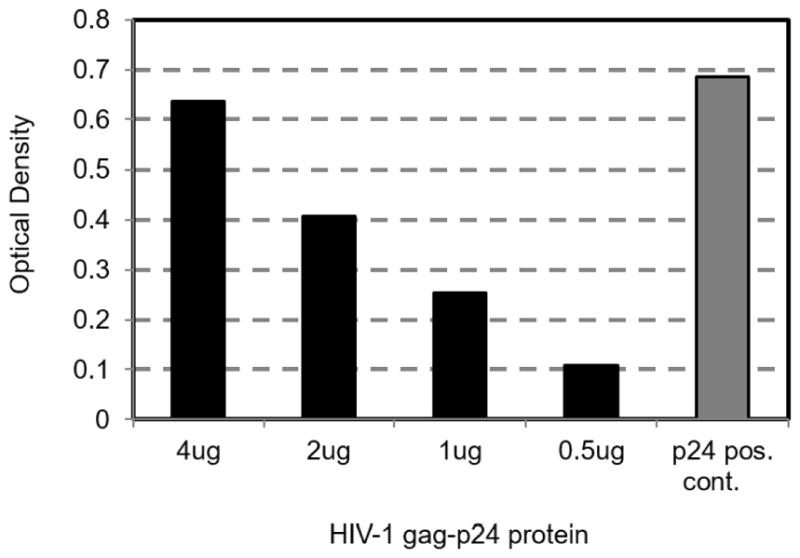
The peptide binds to HIV-1 gag-p24, *in vitro*. ELISA plates were coated with the peptide at 10 μg/ml in coating buffer. Wells were blocked with BSA. Recombinant pure gag p24 was added at decreasing concentrations, as indicated. Following incubation and washings, the complex was probed by mouse anti-gag p24 antibody, followed by anti-mouse conjugate antibody and substrate. Wells coated with gag p24 in the absence of peptide were a positive control. Wells coated with the peptide in the absence of gag p24 were a negative control. The data is representative of 2 independent experiments with similar results.

The 18 amino acid peptide interacts with HIV-1 gag p24. Because the peptide was found in the cytoplasm and nucleus (Figure 1), it is possible that it would interact with one or more of the HIV-1 virus products during or after formation of virus progeny. In this regard, it is known that HIV-1 virus maturation is governed by interactions of HIV-1 gag p24 protein subunits to form the capsid and viable virus progeny. Additional intracellular events, involve formation of the Gag polyprotein in which it interacts with other virus products. To examine whether the peptide could interact with gag p24, an ELISA procedure was developed. Wells were coated with the peptide followed by incubation with purified gag p24. Figure 4 shows specific binding of the peptide to gag p24, revealed by the dose effect. Should similar interaction between the peptide and gag p24 occurs *in vivo*, it would interfere with production of viable HIV-1 progeny.

### LT toxins and their B-subunits synergize with the peptide to increase its activity against HIV-1

**Figure 5:**
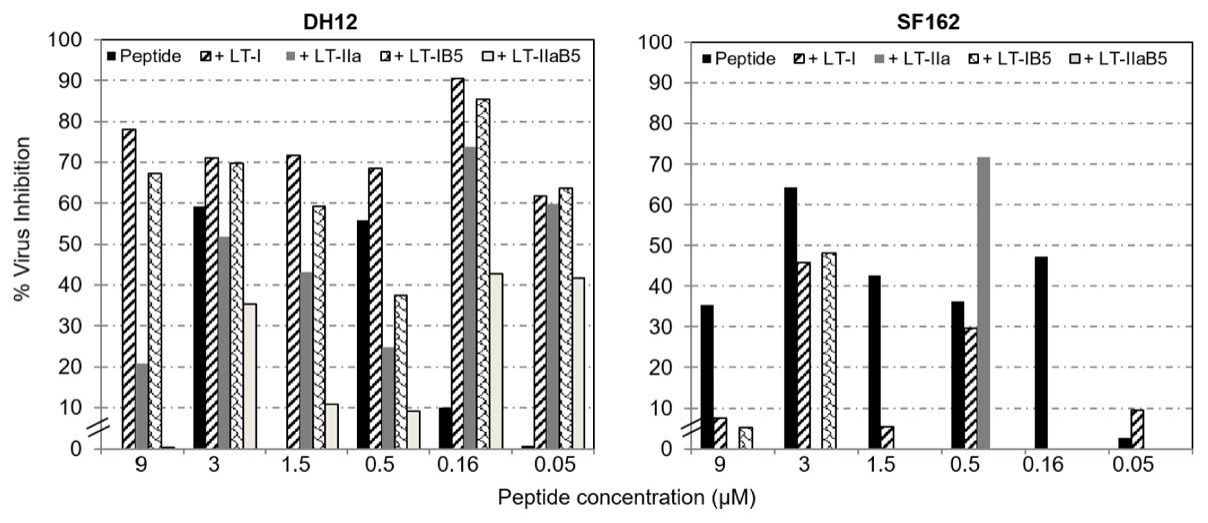
LT toxins and their non-toxic B-subunits synergize with the peptide against HIV-1. Shown are effects of the peptide, toxins or their B-subunits, on virus production and neutralization. 293T cells were seeded in plates for 24 h, transfected with HIV-1 plasmids, and incubated with a mixture of 9 μM of the peptide and LT toxins or their B-subunits (LT-I, 1μg/ml; LT-IIa, 1 μg/ml; LT-IB, 2μg/ml; LT-IIaB, 2μg/ml). The viruses were harvested after 48 h. TZM-bl cells were pretreated with the bacterial toxins or toxoids, for 2 h. Virus-containing supernatants were then serially diluted (5x) in a mixture with increasing amount of the peptide, and added to the cells. Plates were incubated for 48 h, at 37°C. Pates were read to measure the reflective light units in TZM-bl cells, which were converted to “%virus inhibition” relative to a positive control of cells incubated with the virus without the peptide, toxins or their B-subunits. The activity of the peptide, peptide+toxins or their B-subunits, is representative of 2 similar experiments.

To examine whether the peptide activity against HIV-1 strains could be boosted further, the protocol described above for the simultaneous peptide activity on virus production and neutralization was followed, with the exception that the cells were also treated with the toxins before production and just before infection of TZM-bl cells (Figure 5). Treatment with the peptide alone at 9 μM did not inhibit DH12 infectivity. In contrast, treatments with LT-1, LT-IIa, or LT- IB and the peptide neutralized virus infectivity by 78%, 21%, and 67%, respectively. However, LT-IIaB did not have an effect at this dose of the peptide. Similarly, at 1.5 μM, with the exception of the peptide, all toxins and their B-subunits inhibited virus activity by 72%, 43%, 59%, and 11% for LT-I, LT-IIa, LT-IB, and LT-IIaB, respectively. Additionally, at 0.5 μm of the peptide, LT-I, but not the other toxins, inhibited virus infectivity (peptide 56%; +LT-I, 68%). Significantly, at the lower doses of 0.16 and 0.05 μM, the peptide activity against DH12 was boosted by all the toxins and their B-subunits (0.16 μM peptide, 10%; +LT-I, 90%, +LT-IIa, 74%, +LT-IB, 85%, +LT-IIaB, 43%; 0.05 μM peptide, 2%; +LT-I 62%, +LT-IIa 60%, +LT-IB 64%, +LT-IIaB 42%). In comparison with DH12, infectivity of SF162 was neutralized by LT-IIa, but not LT-I or the B- subunits of LT-I and LT-IIa, (0.5 μM peptide, 36%; +LT-IIa, 72%).

We conclude that all the toxins and their B-subunits boost the activity of the 18 amino acid peptide against HIV-1 DH12 production and infectivity, and LT-IIa against HIV-1 SF162, particularly at the low doses of the peptide.

### LT toxins and their B-subunits do not alter cell viability

**Figure 6:**
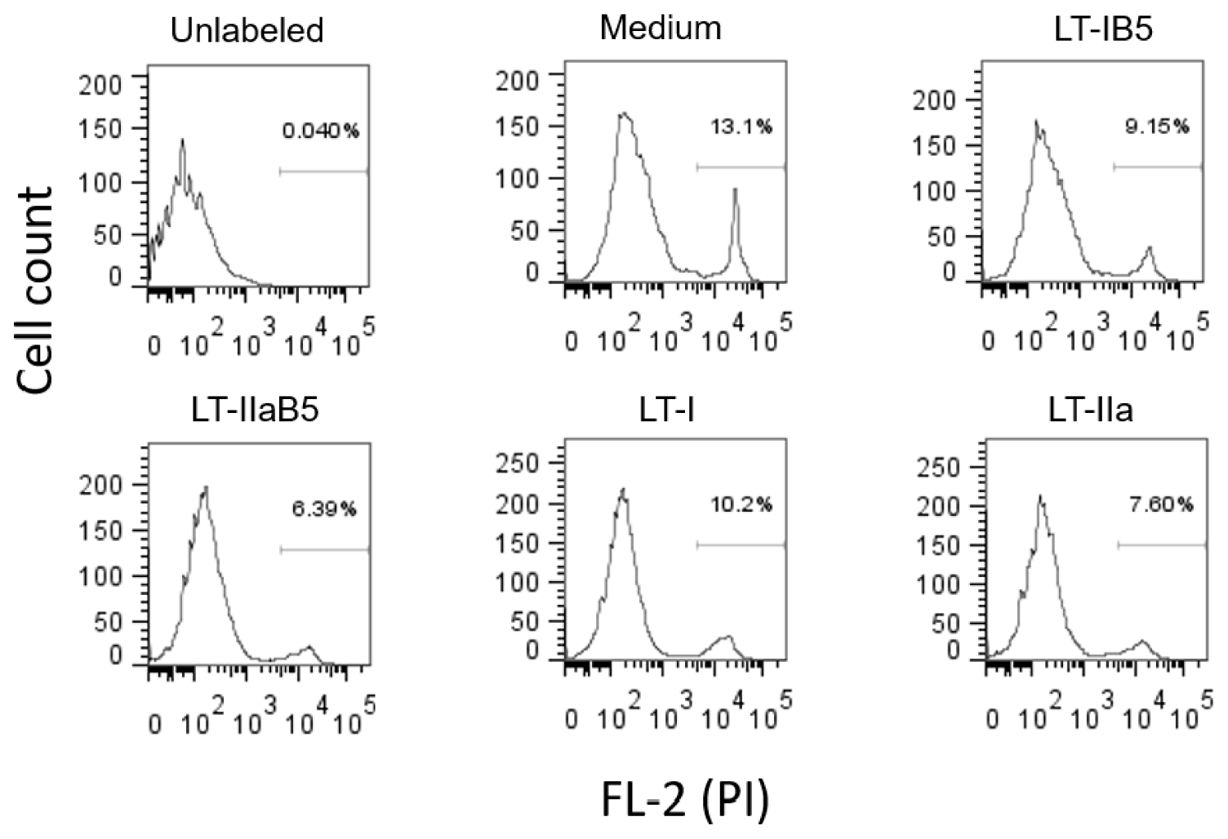
LT toxins and their B-subunits do not alter viability of TZM-bl cells. TZM-bl cells were seeded for 48 h, and incubated with 2 μg/ml LT-IB or LT-IIaB, or 1 μg/ml of each LT-I and LT- IIa, for another 24 h. Following incubation, cells were harvested, washed, and mixed withpropidium iodide. Analysis was performed by flow cytometry. The gate indicates number of cells that are PI^+^.

To exclude the possibility that the toxins or their B-subunits decreased viability of TZM-bl cells, thus resulting in reduction of luminance detection, their viability was examined by flow cytometry with propidium iodide. After 24 h incubation with each of the toxins, no decrease in the viability of these cells was found, compared to control cells incubated in medium only (Figure 6).

## Discussion

In this report, we investigated activity of a cationic peptide against two HIV-1 strains by using an indicator cell line that carries a stably integrated luciferase reporter gene under the control of the HIV-1 regulatory long terminal repeat element. We demonstrate that the peptide inhibits HIV-1 DH12 and SF162 during production and infection in human cell lines. The peptide activity is similar to that of the broadly neutralizing monoclonal antibody 10E8 in that it is more effective in neutralization of DH12 compared to SF162, and had a comparable efficacy against both strains at the high dose of the peptide. The peptide was also effective against DH12 and SF162 virus production and replication, when added simultaneously during production and before infection. Treatment of the cell lines with LT-I and LT-IIa toxins, or their non-toxic B-subunits boosted the peptide activity against HIV-1 DH12 particularly at the low doses of the peptide, and LT-IIa enhanced the peptide activity against HIV-1 SF162. The peptide is shown to cross the cell membrane into the cytoplasm and nucleus, and binds *in vitro* to HIV-1 gag p24, leading to the possibility it may influence stages of HIV-1 particle production and maturation.

The experimental strategy adopted in this work allows rapid evaluation of the amount of HIV-1 infectious virus as a function of a luciferase-activated signal in the reporter cell line [28]. Hence, there is no measurement of non-infectious virus, as it is the case of bulk measurement of extracellular gag p24 in culture supernatants or reverse transcriptase activity [28,38]. The reporter cell line TZM-bl, expresses CD4, CXCR4 and CCR5 receptors; thus, its expression of these receptors is similar to that on the HIV-1 target cell, CD4^+^ T cell, and is suitable for evaluating infection by R5, R4, or dual R5 and R4 HIV-1 viruses (SF162, R5; DH12, dual R5 and R4). In TZM-bl, serum from patients against multiple Env pseudoviruses representing various HIV-1 clades and circulating recombinant forms were investigated [39]. On the other hand, the 293T cells are embryonic kidney epithelial cells competent to replicate vectors carrying the SV40 region of replication, and gives high titers when used to produce retroviruses. Kidney epithelial cells isolated from HIV-1 infected patients are also target of HIV-1 infection [40]. Further, the sensitivity of the pseudovirus assay is higher in comparison with that of uncloned virus produced in peripheral blood mononuclear cells, although this is dependent on both the neutralizing reagent and the virus [41]. Therefore, the pseudovirus assay is well suited to address effects of therapeutic agents on HIV-1 production and replication.

Previously, a peptide of 17 amino acids has been shown to inhibit FIV production by 50%, and impairs infection of CrFK cells, measured by reduction in reverse transcriptase activity [21]. To increase peptide stability, we synthesized an identical peptide with an additional Methionine at its N-terminus. Mapping of the 18 amino acid peptide in the HIdb database [42] revealed an increase in frequency of alpha helix and beta strands, and increased rigidity of the structure compared to the peptide lacking Methionine. In addition to the cationic nature of the peptide, these properties would allow it to enter the lipid bilayer more effectively to reach the cytoplasm and nucleus (Figure 1). Other peptides cross the cell membrane to disrupt HIV-1 replication, or block HIV-1 entry [9,43,44]. Enfuvirtide (T20) is one of the first peptides that has been approved by the FDA, others are currently in clinical trials, while peptides that fail cell entry are modified to penetrate the cell membrane [6,44,45].

Having shown that the 18 amino acids peptide can enter the cell, we hypothesized that it could interfere with HIV-1 production and inhibits HIV-1 replication. Comparative analysis between the peptide and 10E8 antibody confirmed the peptide capacity to impair infection and or/replication of DH12 and SF162 (Figure 2). Interestingly, the kinetics of the peptide and the antibody were similar. Both were more effective in DH12 neutralization compared to SF162, and both attained a neutralization rate of at least 80% at a comparable molarity. Thus, the HIV-1 SF162 gives incrementally lower reverse transcriptase activity compared to other HIV-1 strains, and it is much more difficult to neutralize by 4E10, another monoclonal antibody with a broad activity against HIV-1 [41]. On the other hand, investigation into susceptibility of viruses to peptides revealed significant variability in virus inactivation among different viruses, viruses from the same family, or among different HIV-1 mutant strains [10,39,41]. Small differences in the primary structure of the peptide, the type of virus, and nature of the site of interaction could explain these results. Our own observation supports this view in light of the inability of the 17 amino acid peptide that lacks Methionine to inactivate HIV-1 (not shown) although it inhibited replication of FIV [21]. The results above from neutralization of the virus by the peptide, and the demonstration that some peptides could be dual acting by binding directly and inhibiting assembly of HIV-1 [44] suggest that the 18 amino acid peptide may have impaired virus entry and replication.

Experiments on the effects of the peptide on virus production showed 57% inhibition in the production of infectious HIV-1 DH12 particles. This result is comparable to inhibition of production of FIV, previously reported [21]. However, incubation with the peptide was unable to inhibit production of SF162. Recently, peptides of 12 amino acids, modified to penetrate 293T cells, did not have an effect on the release of a viable HIV-1 molecular clone, despite that high doses of the peptides up to 50 μM were used [44]. Nevertheless, the peptides disrupted HIV-1 Gag processing and resulted in defective infectious particles. In comparison, we used lower doses of our peptide up to 9 μM. It is possible that a higher dose would have an effect on SF162 production. In support, when we added a second dose of the peptide with supernatants from 293 T cells containing SF162, and infected TZM-bl cells with the mixture, it inhibited SF162 infectivity by 64% (Figure 3). Moreover, all doses of the peptide used in the second treatment inhibited SF162. This suggests that repeat treatments or a higher dose of the peptide will likely inactivate both DH12 and SF162, a highly desirable outcome for a therapeutic agent. The possible mechanisms by which cationic peptides inhibit envelope viruses, such as HIV-1, include disruption of viral glycoprotein functions involved in receptor binding and fusion to the cell membrane, alteration of receptor conformation, receptor downregulation, disruption of reverse transcription, and inhibition of virus maturation [9,11,46,47]. Inhibition of maturation occurs by disrupting the final stage of Gag processing, including intermolecular interface interactions of the capsid proteins (Gag and CA) [47].

To understand further the events that mediate our peptide inhibition of HIV-1 virus production, we compared the 18 amino acid peptide sequence to those of HIV-1 proteins available in the NCBI protein database. The query in the database revealed several hits in Gag polyprotein, Gag p24 (CA), reverse transcriptase, and VPU, showing identity that varied between 50–100% (7–8 amino acids). In this regard, several studies highlighted the importance of Gag processing and Gag p24 as targets for drugs or peptides that inhibit HIV-1 particle maturation [13]. Our analysis of Gag p24 and the peptide in an ELISA revealed specific interaction of these molecules (Figure 4). Of note, the recombinant p24 we produced from *E. coli* is a trimer whereas the native protein is a hexamer. Formation of the virus capsid involves assembly of monomers forming two trimers in a hexameric molecule found in the mature core of the virus [48]. Interestingly, drugs that inhibits HIV-1 particle maturation appear to intercalate between the trimers or interfere with CA-CA interaction [13]. Thus, it is possible that our peptide inhibits formation of the capsid by its interaction with the trimer molecule. Further studies will elucidate the nature of such interaction and its impact on HIV-1 Gag processing.

The data on the simultaneous effects of the peptide on production and replication (Figure 3) showed that inactivation of DH12 and SF162 attained a maximum of 64%. We hypothesized that a class of bacterial toxins and toxoids would synergize to boost further the peptide’s activity against HIV-1. Thus, simultaneous treatments of 293T and TZM-bl cells with a mixture of the peptide and each of the bacterial toxins or toxoids significantly boosted the peptide activity against DH12 by 9 and 6 folds at the low doses of 0.16 and 0.05 μM of the peptide, respectively. The latter doses of the peptide alone did not inhibit or minimally inhibited DH12 virus. In comparison, LT-IIa boosted the peptide response against SF162 by 2 folds whereas LT-I or the LT-IB and LT-IIaB toxoids did not have an effect. The inhibitory effects of the holotoxins LT-I and LT-IIa on DH12, and LT-IIa on SF162, could involve cAMP signaling, as it has been shown for cholera toxin, in a human colorectal cell line [16]. However, LT-IB and LT-IIaB, which do not signal for cAMP also boosted the peptide activity against HIV-1 DH12 (Figure 5).

Other possible mechanisms that block HIV-1 infectivity could involve binding of the toxins directly to the virus, blocking site of HIV-1 interaction with its receptors, altering level of receptor expression on TZM-bl, or changing conformation of CXCR4 and CCR5 receptors due to cell membrane capping by the toxins [11,46,47,49,50]. Capping by the toxins results in detergent resistant lipid raft domains, previously shown to bring together signaling molecules and result in intracellular signals [49–54]. However, HIV-1 entry into the cell does not appear to require HIV-1 receptors present in lipid domains; entry was independent of CCR5 and CD4 localization in membrane domains [55]. Hence, it is unlikely that inhibition of HIV-1 was due to a change in the conformation of HIV-1 receptors by the bound toxins. On the other hand, it is possible that the toxins induce intracellular signals that facilitate peptide entry into the cell. Based on the capacity of the B-subunits to induce signals other than cAMP [51,52,54], we suggest that these could influence the HIV-1 cell cycle and its cellular infectivity. Future studies will investigate the mechanistic basis of these observations.

In conclusion, we demonstrate the efficacy of an 18 amino acid peptide against two strains of HIV-1 virus. The peptide inhibited virus production and neutralized virus infectivity in human cell lines, attaining an efficacy against HIV-1 similar to that of the broadly neutralizing monoclonal antibody 10E8, at least against HIV-1 DH12 and SF162 strains. A class of oligomeric toxins further boosted activity of low doses of the peptide against HIV-1. We suggest that the synergy between peptides and bacterial toxins could be advantageous in the fight against HIV-1 infection.
